# Suicidal ideation and attempts in brain tumor patients and survivors: A systematic review

**DOI:** 10.1093/noajnl/vdad058

**Published:** 2023-05-12

**Authors:** Mohammad Mofatteh, Mohammad Sadegh Mashayekhi, Saman Arfaie, Yimin Chen, Armaan K Malhotra, Mohammed Ali Alvi, Nicholas Sader, Violet Antonick, Mostafa Fatehi Hassanabad, Alireza Mansouri, Sunit Das, Xuxing Liao, Roger S McIntyre, Rolando Del Maestro, Gustavo Turecki, Aaron A Cohen-Gadol, Gelareh Zadeh, Keyoumars Ashkan

**Affiliations:** School of Medicine, Dentistry and Biomedical Sciences, Queen’s University Belfast, UK; Neuro International Collaboration (NIC), London, UK; Faculty of Medicine, University of British Columbia, Vancouver, British Columbia, Canada; Neuro International Collaboration (NIC), Vancouver, British Columbia, Canada; Department of Neurology and Neurosurgery, McGill University, Montreal, Quebec, Canada; Department of Molecular and Cell Biology, University of California Berkeley, California, USA; Neuro International Collaboration (NIC), Montreal, Quebec, Canada; Department of Neurology, Foshan Sanshui District People’s Hospital, Foshan, China; Neuro International Collaboration (NIC), Foshan, China; Division of Neurosurgery, Hospital for Sick Children, Toronto, Ontario, Canada; Division of Neurosurgery, Department of Surgery, University of Toronto, Toronto, Ontario, Canada; Department of Neurologic Surgery, Mayo Clinic, Rochester, MN, USA; Neuro International Collaboration (NIC), Toronto, Ontario, Canada; Division of Neurosurgery, University of Calgary, Alberta, Canada; Neuro International Collaboration (NIC), Calgary, Alberta, Canada; University of Vermont, Burlington, Vermont, USA; Neuro International Collaboration (NIC), Vermont, USA; Division of Neurosurgery, University of British Columbia, Vancouver, British Columbia, Canada; Department of Neurosurgery, Penn State Milton S. Hershey Medical Center, Hershey, Pennsylvania, USA; Division of Neurosurgery, St. Michael’s Hospital, Toronto, Ontario, Canada; Department of Neurosurgery, Foshan Sanshui District People’s Hospital, Foshan, China; Department of Surgery of Cerebrovascular Diseases, Foshan First People’s Hospital, Foshan, China; Neuro International Collaboration (NIC), Toronto, Ontario, Canada; Mood Disorder Psychopharmacology Unit, University Health Network, University of Toronto, Toronto, Ontario, Canada; Department of Psychiatry, University of Toronto, Toronto, Ontario, Canada; Brain and Cognition Discovery Foundation, Toronto, Ontario, Canada; Neuro International Collaboration (NIC), Montreal, Quebec, Canada; Neurosurgical Simulation and Artificial Intelligence Learning Centre, Department of Neurology & Neurosurgery, Montreal Neurological Institute and Hospital, McGill University, Montreal, Quebec, Canada; McGill Group for Suicide Studies, Douglas Hospital Research Center, Montreal, Canada; Department of Psychiatry, McGill University, Montreal, Quebec, Canada; The Neurosurgical Atlas, Carmel, Indiana, USA; Department of Neurological Surgery, Indiana University, Indianapolis, Indiana, USA; Neuro International Collaboration (NIC), Indiana, USA; Division of Neurosurgery, Department of Surgery, University of Toronto, Toronto, Ontario, Canada; Neuro International Collaboration (NIC), London, UK; Department of Neurosurgery, King’s College Hospital NHS Foundation Trust, London, UK; Department of Basic and Clinical Neuroscience, Institute of Psychiatry, Psychology and Neuroscience, King’s College London, UK; King’s Health Partners Academic Health Sciences Centre, London, UK; School of Biomedical Engineering and Imaging Sciences, Faculty of Life Sciences and Medicine, King’s College London, UK

**Keywords:** brain tumor, depression, glioblastoma, mental health, suicide

## Abstract

**Background:**

Subsequent to a diagnosis of a brain tumor, psychological distress has been associated with negative effects on mental health as well as suicidality. The magnitude of such impact has been understudied in the literature. We conducted a systematic review to examine the impact of a brain tumor on suicidality (both ideation and attempts).

**Methods:**

In accordance with the PRISMA guidelines, we searched for relevant peer-reviewed journal articles on PubMed, Scopus, and Web of Science databases from inception to October 20, 2022. Studies investigating suicide ideation and/or attempt among patients with brain tumors were included.

**Results:**

Our search yielded 1,998 articles which were screened for eligibility. Seven studies consisting of 204,260 patients were included in the final review. Four studies comprising 203,906 patients (99.8%) reported elevated suicidal ideation and suicide attempt incidence compared with the general population. Prevalence of ideation and attempts ranged from 6.0% to 21.5% and 0.03% to 3.33%, respectively. Anxiety, depression, pain severity, physical impairment, glioblastoma diagnosis, male sex, and older age emerged as the primary risk factors associated with increased risk of suicidal ideation and attempts.

**Conclusion:**

Suicidal ideation and attempts are increased in patients and survivors of brain tumors compared to the general population. Early identification of patients exhibiting these behaviors is crucial for providing timely psychiatric support in neuro-oncological settings to mitigate potential harm. Future research is required to understand pharmacological, neurobiological, and psychiatric mechanisms that predispose brain tumor patients to suicidality.

**Key Points**

This systematic review showed that suicidal ideation and attempts are increased in patients and survivors of brain tumors compared to the general population. This is the first systematic review on suicidal ideation and attempt among brain tumor patients.

**Importance of the Study**

Diagnosis and treatment of brain tumors can be associated with increased stress, anxiety, and depression among patients. In severe cases, mental health problems can be manifested as suicidality in brain tumor patients and survivors. By reviewing published literature, this study showed that suicidal ideation and attempts are increased in patients and survivors of brain tumors compared to the general population. Identifying patients with suicidality is crucial for providing timely psychiatric support in neuro-oncological settings to mitigate potential harm. Holistic follow-up care, including psychiatric risk assessment and support is required for brain tumor patients. Highlighting such problems can help identify limitations, tackle challenges, and facilitate future research to improve brain tumor patients’ psychiatric outcomes. This is the first systematic review of published literature on suicidal ideation and attempt among brain tumor patients.

Cancer is the second cause of death globally with an adverse impact on years of life lost, years lived with disability, and disability-adjusted life years.^1^ Increased stress, anxiety, and depression have been identified as postcancer diagnosis sequelae, especially among patients with brain tumors.^2–4^ Existing evidence has shown a trend toward increased suicidality in cancer patients compared to the general population,^5,6^ particularly in patients with physical impairments and poor prognoses.^7,8^ Suicidal ideation (SI) and suicidal attempt (SA) are two manifestations of extreme psychological distress.^9^ SI is understood as purposeful thinking or planning of potential measures for self-harm tendencies and ending one’s life.^10^ SI is also frequently used as a reliable clinical predictor for future SA and behaviors.

Suicide has become a major public health concern globally and one of the leading causes of death.^11^ In 2019, there were 45,861 deaths from suicide in the United States, along with 381,295 emergency department visits for non-fatal self-inflicted injuries.^11^ As a complex biopsychosocial behavior, suicide is associated with multiple factors, including genetic susceptibility, biological diseases, psychological disorders, and social circumstances.^12,13^ Suicide among cancer patients is likely multifactorial in part related to severe psychological distress during treatment and hopelessness induced by loss of control.^14–18^ Furthermore, increased predilection toward suicidality can be based on brain pathways affected by tumor location and surgical routes.^19–21^

The first case of suicidality related to central nervous system cancer was described in a patient with cerebral neuroblastoma in 1989.^22^ Since then, the association between brain tumors and suicide remains understudied with sparse available literature.^23^ Deaths attributable to suicide represent preventable and potentially actionable mortalities if appropriate identification and risk stratification are implemented. The purpose of this systematic review is to characterize SI and SA in brain tumor patients, assess the magnitude of this association, and raise awareness among clinicians and healthcare providers.

## Materials and Methods

### Search Strategy

We conducted this systematic review in accordance with the Preferred Reporting Items for Systematic Reviews and Meta-Analysis (PRISMA) guidelines^24^ to identify published literature reporting on SI and SA among brain cancer patients. Cancer and suicides were respectively defined based on the International Classification of Disease (ICD)-10 codes C00-C97 (malignant neoplasms, X60-X84 (intentional self-harm) and Y87.0 (intentional self-harm sequelae).^25^ The electronic search terms were carried out using PubMed, Scopus, and Web of Sciences from inception to October 20, 2022 for relevant articles. The following Boolean terms were used for the search in different combinations: (“brain tumor” OR “brain cancer” OR “cranial tumor” OR “cranial cancer” OR “intra-cranial tumor” OR “intra-cranial cancer” OR “brain metastasis,” etc.) AND (“suicide” OR “self-harm” OR “self-injury,” etc.) (**Supplementary Table 1**).

### Inclusion and Exclusion Criteria

**Figure 1.**
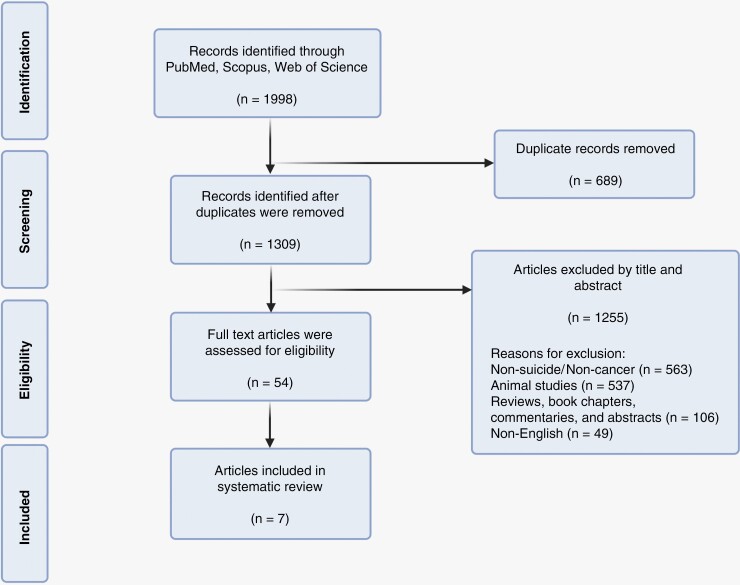
Preferred Reporting Items for Systematic Reviews and Meta-Analyses (PRISMA) flowchart demonstrating search, screen, inclusion, and exclusion process for the current study

We considered papers eligible for our systematic review if they met the following conditions: (1) original articles, (2) published in English only, (3) investigated SI or SA, (4) exclusively reported on human subjects, and (5) data could be extracted on brain tumor patients and/or survivors. The exclusion criteria were defined as (1) studies that investigated pathologies other than brain cancer, (2) studies investigating SI and SA in mixed cancer populations where sufficient data on brain cancer patients were not provided (e.g. Recklitis et al.;^26^ Lu et al.;^27^ Gunnes et al.;^28^ Osazuwa-Peters et al.;^29^ Sharkey et al.;^30^ Storm et al.^31^), (3) case studies, and (4) studies which investigated psychiatric conditions other than SI and SA. Based on these specified criteria, title and abstract screening were performed after initial duplicate removal (Figure 1). Full-text articles were assessed for eligibility. Three authors (M.M., M.S.M., and S.A.) screened relevant articles from the reference lists of selected articles to ensure no additional relevant articles were excluded.

### Data Extraction

The following data were extracted from the final articles: first author, publication year, title, journal, country, study objectives, study type, study period, study design (single/multi-center), sample size, sex and ethnicity of participants, mean, range, and standard deviation of age years at diagnosis and follow-up, pediatric versus adult cohort (>18 years), and time since diagnosis. Brain tumor-specific details included the number of patients with differing cancer subtypes, brain hemisphere, anatomic location, treatment details, resection extent, and whether participants were treated or actively undergoing therapy were also extracted. Psychiatric details were identified, including the number of patients with SI and SA, comorbidities and complications, psychiatric comorbidity, psychiatric factors screened and assessment method utilized, risk factors reported, main outcomes, and whether higher risk of SI or SA was reported in comparison to a reference population in each study. All calculations were done on Microsoft Excel (version 2016; Microsoft, Redmond, WA, USA).

## Results

The search strategy resulted in 1,998 articles across PubMed (*n* = 880), Scopus (*n* = 879), and Web of Science (*n* = 239). Duplicates (*n* = 689) were removed. The remaining studies (*n* = 1,309) were screened based on their titles and abstracts, and irrelevant articles were removed (*n* = 1,255). The remaining articles (*n* = 54) were subject to full-text review to determine inclusion based on established eligibility criteria. Seven studies met our criteria and were included in the final review (Figure 1).

### Overview of Included Studies

A summary of included articles is shown in Table 1. Five studies (71.4%) reported patient cohorts from the USA,^32–35^ one (14.3%) from Lithuania^37^ and another (14.3%) from Germany.^38^ Four included studies (57.1%) were retrospective,^32,33,35,36^ and three (42.9%) were prospective.^34,37,38^

**Table 1.** An Overview of Studies Included in the Final Review

Study	Title	Journal	Country	Objective
**Turner et al.** ^32^	Medical, psychological, cognitive and educational late-effects in pediatric low-grade glioma survivors treated with surgery only	Pediatric Blood & Cancer	USA	To describe the multidimensional late-effects of pediatric LGG survivors treated exclusively with surgery.
**Brinkman et al.** ^33^	Suicide ideation in pediatric and adult survivors of childhood brain tumors	Journal of Neuro-Oncology	USA	To report on suicide ideation in a sample of youth and adult survivors of pediatric brain tumors.
**Lucas et al.** ^34^	Assessing suicidal ideation and behaviors among survivors of childhood brain tumors and their mothers during socio-behavioral research	Oncology Nursing Forum	USA	To describe the development and feasibility of a protocol for nonpsychiatric subspecialty research staff members to screen suicidal ideations or behaviors among adolescent and young adult survivors of childhood brain tumors and their mother caregivers during data collection.
**Hickmann et al.** ^38^	Suicidal ideation, depression, and health-related quality of life in patients with benign and malignant brain tumors: a prospective observational study in 83 patients	Acta Neurochirurgica	Germany	To investigate the prevalence of SI, depression, and their association with HRQoL in patients with intra- and extra-axial tumors during the first nine months after diagnosis.
**Pranckeviciene et al.** ^37^	Suicidal ideation in patients undergoing brain tumor surgery: prevalence and risk factors	Support Care Cancer	Lithuania	To investigate the prevalence rate and correlation of pre-operative SI in brain tumor patients admitted for elective surgery.
**Saad et al.** ^35^	Association of brain cancer with risk of suicide	JAMA Network Open	USA	To investigate the increase in suicide rate associated with a diagnosis of brain cancer.
**Zhou et al.** ^36^	Incidence, trend and risk factors associated with suicide among patients with malignant intracranial tumors: a surveillance, epidemiology, and end results analysis	International Journal of Clinical Oncology	USA	To investigate the suicide rates and identify risk factors for suicide among patients with malignant intracranial tumors.

HRQoL, health-related quality of life; LGG, low-grade glioma; SI: suicide ideation; USA, United States of America.

### Baseline Characteristics of Neuro-oncological Cohorts

Overall, 204,260 patients were included across seven studies, of which 114,299 patients (56.0%) were males, and 89,961 patients (44.0%) were females. There was a heterogeneous sample size ranging from 60 patients^32^ to 115,668 patients.^36^ Two large epidemiological studies draw data from the Surveillance, Epidemiology, and End Results (SEER) Program, and they included overlapping time periods.^35,36^ More specifically, Zhou et al.^36^ study extracted data on suicides in patients with brain tumors between 1975 and 2015, and the Saad et al.^35^ analysis covered the years 2000–2016. Therefore, it is possible that some of the same patient data points are included in both studies. Three studies (42.9%) were exclusively conducted on pediatric patients,^32–34^ two (28.6%) had exclusively adults,^37,38^ and two (28.6%) included a mixture of adult and pediatric patients.^35,36^ Four studies (57.1%) reported the age of patients at diagnosis.^32,33,37,38^ The youngest mean age at diagnosis was 6.8 years,^32^ whereas the highest was 55.9 years.^37^ Three studies (42.9%) reported information on the ethnicity of patients:^32,35,36^ 177,012 patients (86.7%) were white, and 14,217 patients (6.96%) were black/African American. The timespan of included studies was 40 years. Zhou et al.^36^ included patient information from 1975 to 2015, and Saad et al.^35^ reported data from 2000 to 2015 (Table 2).

**Table 2.** An Overview of Studies Characteristics and Patients Demographics

Study	Study period	Study design	Single/ multi-center	Sample size	Sex (M, F)	Ethnicity (n, %)	Adult (>18 years) and/or pediatric (n, %)	Mean age at diagnosis ± SD (range)	Mean age at follow-up/interview (SD, range)	Mean time since diagnosis ± SD (range)
**Turner et al.** ^32^	Jan 2003- Sep 2007	Retrospective	Single-center	60	26 (43.3%), 34 (56.7%)	White (51, 85.0%), black/ African American (4, 6.67%), Hispanic (3, 5.0%), other (2, 3.33%)	Pediatric	6.8 y (0.1-19.0)	16.3 y (5.8-34.2)	8.4 y (3.9 - 20.4)
**Brinkman et al.** ^33^	Jan 2003- Sep 2007	Retrospective	Single-center	319	143 (44.8%), 176 (55.2%)	NS	Pediatric	8.0 ± 4.9 y	18.0 ± 4.9 (10-35,)	10 ± 5.0 y
**Lucas et al.** ^34^	NS	Prospective	Single-center	134	105 (78.4%), 29 (21.6%)	NS	Pediatric	NS	20.52 y (5.28, 14-39)	13.12 ± 6.26 y (5-32)
**Hickmann et al.** ^38^	Oct 2010- Dec 2012	Prospective	Single-center	83	36 (43.4%), 47 (56.6%)	NS	Adult	51.9 ± 12.8 y (20.5-76.8)	NA	NA
**Pranckeviciene et al.** ^37^	May 2010- Dec 2011	Prospective	Single-center	211	64 (30.3%), 147 (69.7%)	NS	Adult	55.9 ± 15.4 y	NA	NA
**Saad et al.** ^35^	Jan 2000- Dec 2016	Retrospective	Multi-center	87785	48908 (55.7%), 38877 (44.3%)	White (75616, 86.1%), black (6511, 7.42%), Asian or Pacific islander (5258, 5.99%), other (400, 4.56%)	Adult (76218, 86.8%), and pediatric (11657, 13.3%)	NS	NS	NS
**Zhou et al.** ^36^	1975-2015	Retrospective	Multi-center	115668	65017 (65.2%), 50651 (43.8%)	White (10134, 87.6%), black/ African American (7702, 6.66%), other (6621, 5.72%)	Adult and pediatric	NS	NS	NS

F, female; M, male; NS, not specified; SD, standard deviation; Y, years.

### Neuro-oncological, Neuroanatomical, and Treatment Details

Glioblastoma was the most common cancer type in 92,829 patients (45.4%) in papers that specifically reported glioma subtypes.^35,36,38^ Treatment was reported in five studies (71.4%).^32,33,36–38^ Among them, 78,544 patients underwent surgery (38.5%) as part of their treatment plans. There was a significant statistical association between SI and surgery-only treatment (*p* = 0.04) as well as cranial radiation (*p* = 0.04) reported by Brinkman et al.^33^ (Table 3).

**Table 3.** Disease Pathology and Treatment Overview in Included Studies

Study	Cancer type (n, %)	Cancer hemisphere (n, %)	Cancer location (n, %)	Treatment (n, %)	Resection extent (n, %)
**Turner et al.** ^32^	LGG (60, 100%)	NS	Frontal (5, 8.33%), parietal (2, 3.33%), temporal (13, 21.7%), Posterior fossa/ cerebellum (28, 46.7%), intraventricular (4, 6.67%), thalamus (3, 5.0%), hypothalamus/ suprasellar (2, 3.33%), optic nerve/ pathway (2, 3.33%), cervicomedullar (1, 1.67%), pineal (1, 1.67%)	Surgery (60, 100%)	Total/ near total (40, 66.7%), Subtotal (15, 25.0%), biopsy only (5, 8.33%)
**Brinkman et al.** ^33^	LGG (162, 50.8%), PNET/medulloblastoma (64, 20.1%), germ cell tumor (28, 8.8%), craniopharyngioma (23, 7.2%), ependymoma (14, 4.4%), other (NS) (28, 8.8%)	NS	Posterior fossa/cerebellum (110, 34.5%), diencephalon/ brain stem (99, 31.0%), cerebral cortex (110, 34.5%)	Surgery (99, 31.0%), Surgery & radiation (95, 29.8%), Surgery, radiation & chemotherapy (84, 26.3%), other (NS) (41, 12.9%)	Total (50, 15.7%), subtotal/ near total (31, 9.72%), none/ biopsy (15, 4.70%)
**Lucas et al.** ^34^	LGG (102, 76.1%), HGG (7, 5.22%), PNET (57, 42.5%), craniopharyngioma (14, 10.4%), germ cell (6, 4.48%), ependymoma (5, 3.73%), choroid plexus (5, 3.73%), other (7, 5.22%)	NS	Posterior fossa (104, 77.6%), cortical (35, 26.1%), sellar (29, 21.6%), pineal (8, 6.0%), thalamic (8, 6.0%), ventricle (7, 5.2%), brain stem (5, 3.7%), optic nerve (4, 3.0%)^*^	NS	NS
**Hickmann et al.** ^38^	Meningioma (32, 38.5%), glioblastoma (24, 28.9%), astrocytoma (6, 7.23%), anaplastic astrocytoma (6, 7.23%), hemangioblastoma (2, 2.41%), acoustic neuroma (2, 2.41%), others (11, 13.3%)	Left (32, 38.6%), right (40, 48.2%)	NS	Surgery (83, 100%)	Total/ near total (27, 32.5%), Subtotal (55, 66.3%)
**Pranckeviciene et al.** ^37^	LGG (15, 7.11%), HGG (35, 16.6%), meningioma (82, 38.9%), pituitary adenoma (28, 13.3%), acoustic neuroma (17, 8.06%), other (NS) (34, 16.1%)	NS	NS	Surgery (211, 100%)	NS
**Saad et al.** ^35^	Glioblastoma (39,248, 44.7%), other (NS) (48537, 55.3%)	NS	NS	NS	NS
**Zhou et al.** ^36^	Glioblastoma (53,557, 46.3%), other glioma (40565, 35.1%), other malignancies (NS) (21546, 18.6%)	NS	NS	Surgery (77912, 67.4%), chemotherapy (49646, 42.9%), Radiotherapy (74725, 64.6%)**	NS

HGG, high-grade glioma; LGG, low-grade glioma; NS, not specified; PNET, primitive neuroectodermal tumors.

^*^Patient number exceeded the total sample size of 134. ** It was not specified if this was the only treatment or in combination.

Three studies (42.9%) reported anatomical cancer locations,^32–34^ and one study (14.3%)^38^ specified the brain tumor hemisphere. Patients with supratentorial tumors were more likely to commit suicide compared to patients with infratentorial tumors (*p* = 0.018) (Table 3).^36^ This observation was considered by reporting authors to reflect a propensity for supratentorial compared to infratentorial lesions to cause neurological deficits affecting psychological aberration, mood dysregulation, and social impairments.^36^

### Suicide Ideation and Attempt Details

Three papers (42.9%) investigated SI and SA among brain cancer survivors,^32,33^ one paper (14.3%) examined brain cancer patients,^37^ and one (14.3%) reported on a mixture of patients and survivors.^38^ Two studies (28.6%) did not specify whether their samples were patients with brain neoplasm patients and/or survivors who had recovered (Table 4).^35,36^

**Table 4.** Suicide Ideation, Attempt, and Psychiatric Overview of Patients and/or Survivors

Study	Patients and/ or survivors	Suicide ideation (n, %)	Suicide attempt (n, %)	Comorbidity/ complication (n, %)	Psychiatry comorbidity (n, %)	Psychiatric factors screened	Psychiatric assessment method
**Turner et al.** ^32^	Survivors	9 (15.0%)	2 (3.33%)	Hydrocephalus (34, 56.7%), motor dysfunction (26, 43.3%), visual problems (19, 31.7%), social difficulties (19, 31.7%), Seizure/epilepsy (15, 25.0%)	Anxiety (11, 18.3%), depression (9, 15.0%)	Anxiety and depression	Semi-structured interview developed by the team based on the DSM IV
**Brinkman et al.** ^33^	Survivors	37 (11.7%)	5^*^ (1.57%)	Hydrocephalus (114, 51.4%), seizures (53, 16.6%), neurofibromatosis (21, 6.6%), posterior fossa syndrome (14, 4.4%), disease recurrence/ progression (75, 23.6%)	Depression (130, 40.8%), anxiety (88, 27.6%), social problems (147, 46.1%), behavior problems (70, 21.9%), psychoactive medications (72, 23.7%)	Anxiety and depression	Semi-structured clinical interview based on DSM IV
**Lucas et al.** ^34^	Survivors	11 (8.2%)	NS	NS	NS	NS	BSI, C-SSRS
**Hickmann et al.** ^38^	Patients and survivors	17 (21.5%)	0 (0%)	NS	Patients with existing depression were excluded	Depression	BDI, EORTC QLQ- C30/BN20
**Pranckeviciene et al.** ^37^	Patients	12 (6%)	NS	NS	History of psychiatric comorbidity (15, 7.11%)	Anxiety and depression	BDI-II, HADS, SF-36 scale, Barthel Index
**Saad et al.** ^35^	NS	NS	29**(0.03%)	NS	NS	NS	NS
**Zhou et al.** ^36^	NS	NS	99** (0.086%)	NS	NS	NS	NS

BDI, Beck depression inventory; BSI, brief symptom inventory; C-SSRS, Columbia-suicide severity rating scale; DSM, diagnostic and statistical manual of mental disorders; EORTC QLQ- C30/BN20, The European organization for research and treatment of cancer quality of life questionnaire; HADS, hospital anxiety and depression scale; NS, not specified; SF-36, short form survey.

^*^Brinkman and colleagues^33^ reported no fatal attempts in their study. **Zhou et al.^36^ and Saad et al.,^35^ reported completed suicide attempts only but did not specify the number of attempts not resulting in death.

Three studies (42.9%) reported both SI and SA,^32,33,38^ two studies (28.6%) focused only on SI,^34,37^ and two other studies (28.6%) investigated SA.^35,36^ The range of SI rates reported was 6.0%^37^ to 21.5%^38^ (Table 4). Authors speculated that the low SI rate by patients investigated by Pranckeviciene et al.^37^ was due to the timing of neuropsychological assessment as the study was carried out before patients were treated with neurosurgery (in the hyperacute postdiagnosis period). Four studies (57.1%) screened for psychiatric factors and demonstrated anxiety and depression were elevated among brain tumor patients.^32,33,37,38^ Brinkman et al.^33^ reported that 37 patients (11.7%) out of the total 319 pediatric brain tumor patients had SI and five patients (1.6%) had documented SA. In their samples, similar to other studies,^34^ history of depression was the strongest predictor of SI.^33^ While none of these SA resulted in fatalities, four patients (1.3%) were admitted to inpatient psychiatric care units. All five patients who attempted suicide had a history of depression. The same study showed a significant statistical association between SI and depression (*p* < 0.001), anxiety (*p* < 0.001), and psychoactive medication (*p* < 0.001) (Table 4).^33^

Turner et al.^32^ reported that 75% of cancer survivors out of their 60 patients had significant psychological challenges since their diagnosis; the most common psychosocial issues were anxiety (*n* = 29, 48.3%), depression (*n* = 22, 36.7%), social challenges (*n* = 19, 31.7%), and behavioral problems (*n* = 17, 28.3%). There was evidence of differences in these measures when compared to national mental health registry data for pediatric populations (*p* = 0.05) and adult populations (*p* = 0.002). Mood disorders in low-grade glioma (LGG) survivors who underwent surgery alone were elevated threefold compared to the general population (*p* = 0.018). Among LGG survivors post-surgery, 72% had ongoing complications, such as motor dysfunction, ataxia, vision difficulties, speech problems, endocrinopathies, and seizures, years after their surgery^32^ (Table 4).

Pranckeviciene and colleagues^37^ reported that anxiety symptoms (*p* = 0.01), physical impairments (*p* = 0.01), pain severity (*p* = 0.03), worsening mental health (*p* < 0.01), and past history of psychiatric disorders (*p* = 0.03) were associated with increased risk of SI. In their sample, 87% of brain tumor patients with SI had a history of depression (Table 4).^37^ While some studies excluded patients with pre-existing depression,^38^ others did not specify whether baseline mental health conditions were controlled or excluded. Some studies did not quantify the severity of each described physical and psychological issue (Table 4).

### Factors Associated Sith Suicide Ideation and Attempt Among Brain Tumor Patients

Different studies reported various risk factors that could elevate the risk of SI and SA. Older age (60–79 years vs. ≤ 39 years, *p* = 0.002), male sex (*p* < 0.001), glioblastoma diagnosis (other gliomas vs. glioblastoma, *p* = 0.022; other malignancy vs. glioblastoma, *p* = 0.009) and supratentorial tumor (infratentorial vs. supratentorial, *p* = 0.009) were associated with increased risk of suicide among a large sample of 115,668 American patients studied between 1975 and 2015.^36^ Another study corroborated these findings by demonstrating that male sex (observed (*O*)/expected (*E*), 3.38), age older than 64 (O/E, 5.04), and glioblastoma diagnosis (*O/E*, 4) were associated with increased risk of suicide.^35^ Similarly, Zhou et al.^36^ demonstrated that the majority (*n* = 87, 88%) of all SAs (*n* = 99) occurred in males with brain tumors. Brinkman et al.^33^ also reported a significant association between older age at diagnosis (*p* = 0.017) and follow-up in the neuro-oncology clinic (*p* = 0.007) with SI among pediatric survivors of brain tumors. In contrast, there was no statistical significance between SI and sex (*p* = 0.57), tumor location (*p* = 0.30), time since diagnosis (*p* = 0.76), and disease recurrence/progression (*p* = 0.56) in their sample (Table 5).

**Table 5.** An Overview of Risk Factors Identified and Main Outcomes Reported

Study	Risk factors identified	Main outcomes reported	Limitation	Higher SI/SA risk compared to the general population (*p-value*)/ General population source
**Turner et al.** ^32^	NS	Surgery-only LGG survivors may be more affected by their tumor and its resection than previously appreciated. Mental health problems were elevated compared to the general population for both older children (*p* = 0.05) and adults (*p* = 0.002). SI was similar compared to non-CNS childhood cancers	Small sample size, retrospective design, single-center study	NS
**Brinkman et al.** ^33^	Sex, age, and history of depression (*OR* = 20.6, 95 % *CI* = 4.2–101.1), psychoactive medication treatment (*OR* = 4.5, 95 % CI = 1.8–11.2), observation or surgery-only treatment (*OR* = 3.7, 95% *CI* = 1.5–9.1), and seizures (*OR* = 3.6, 95% *CI* = 1.1–11.1) were significantly associated with SI in survivors.	Survivors of pediatric brain tumors appear to be at risk for experiencing SI. A significant number of pediatric brain survivors (11.7%) reported SI, which is higher than the 12 months SI prevalence of 3.7% in the US general population. History of depression is the strongest predictor of SI.	Retrospective design, single-center study	Yes (NS)/ General US adult population^37^
**Lucas et al.** ^34^	NS	Survivors of childhood brain tumors and their caregivers may experience psychosocial distress. SI rates are higher than the 12-month suicidal ideation prevalence in adults (4%) in the general population of the USA. Nurses, as research assistants or in other roles, can use tools such as the C-SSRS to assist in front-line assessments.	Small sample size, single-center study	Yes (NS)/ General US adult population^37^
**Hickmann et al.** ^38^	NS	Patients with intracranial tumors have decreased HRQoL and SI regardless of histopathology. SI is associated with higher BDI scores, but no evident depression (BDI ≥ 18).	Small sample size, heterogenous sample	NS
**Pranckeviciene et al.** ^37^	Severity of anxiety symptoms (*p* = 0.01), physical impairment causing role limitations (*p* = 0.01), pain severity (*p* = 0.03), worse mental health (*p* < 0.01), and a past history of psychiatric disorders (p = 0.03)	SI was present in 6 % of patients before surgical intervention and was associated with a history of psychiatric disorders and worse perceived health status.	Small sample size, heterogenous sample	NS
**Saad et al.** ^35^	Male sex (*O/E*, 3.38), age older than 64 (*O/E*, 5.04), and glioblastoma diagnosis (*O/E*, 4)	Patients diagnosed with brain cancers had a higher rate of suicide within the first year after their diagnosis compared with the general population.	Retrospective study, heterogenous sample	Yes (NS)/ US general population
**Zhou et al.** ^36^	Male sex (*SMR* = 1.78), older age (60 - 79 years, *SMR* = 3.54), white ethnicity (*SMR* = 1.86), being married (*SMR* = 2.31), living in rural areas (*SMR* = 2.50), history of other malignancy (*SMR* = 3.81), diagnosis of glioblastoma (*SMR* = 4.05) and supratentorial location (*SMR* = 2.45).	Male sex, older age, and supratentorial location were significantly associated with increased risk of SA, especially within the first year following diagnosis.	Retrospective study, heterogenous sample	Yes (*p* < 0.001)/ US general population

BDI, Beck depression inventory; CI, confidence interval; HRQoL, health-related quality of life; LGG, low-grade glioma; O/E: observed to expected; SI, suicide ideation; SMR, standardized mortality ratio; US, United States.

Ideation and attempt of suicide can be elevated at specific phases of neuro-oncological diagnosis and treatment. Hickman et al.^38^ showed the highest rate of SI was present in the sixth- and ninth-months following surgery. Patients with SI had higher Beck’s Depression Index (BDI) scores compared to patients without SI at three (*p* < 0.031) and six (*p* < 0.001) months post-surgery. Similarly, a large-scale retrospective study of 115,668 patients demonstrated the suicide rate was highest within the first year after diagnosis (standardized mortality ratio (SMR), 13.04) and decreased afterward with a similar rate to the general population five years post-diagnosis (SMR, 0.88).^36^ Moreover, brain tumor patients diagnosed with severe depression (BDI ≥ 18) had significantly worse global health status (*p* = 0.001), emotional status (*p* = 0.004), cognitive function (*p* = 0.001), and future uncertainty (*p* = 0.009) at nine months post-surgery^38^ (Table 5).

Socioeconomic factors have been shown to influence the prevalence of suicide.^11,39^ Education level (*p* = 0.10) and marital status (*p* = 0.10) were not associated with an increased risk of SI.^37^ Similarly, other studies reported that there were no significant differences in the occurrence of SI based on religion, education level, and native language (German vs non-German); however, the level of statistical significance and p-values were not reported.^38^ Ethnicity (*p* = 0.08), marital status (*p* = 0.19) and high poverty (*p* = 0.367), frontal lesion (*p* = 0.777), and history of other malignancy and treatment (*p* = 0.131), were not associated with the risk of suicide.^36^ Brain tumor survivors treated with psychoactive medications were more likely to experience SI (*p* < 0.001).

## Discussion

Suicide is one of the leading causes of death worldwide.^41,42^ Epidemiological studies indicate that the lifetime prevalence of suicide attempts is 4.1% for adolescents and 0.6% for adults.^43^ Suicide can be correlated with poor physical and mental health,^44^ and cancer patients can experience psychological distress resulting in SI/SA.^30^ The rate of SI in patients diagnosed with cancers (such as prostate cancer and adult survivors of childhood cancer) is higher than in comparable population baseline; however, till date, the SI and SA rates among central nervous system tumor populations have not been well described.^26,30,45^

The rate of SI among brain tumor patients and survivors included in this review was higher than the general population prevalence.^40^ While SI and/or SA were elevated compared to baseline population measures, our findings were similar to reported rates among noncentral nervous tumors.^32,44^ Evidence suggests that SI is a reliable predictor of SA.^14^ Clinically, this underscores the importance of early identification of patients with an elevated risk of SI for neuro-oncological healthcare providers.^37^ Additionally, some studies speculated that physical disabilities or communication barriers in patients with late-stage brain tumor prognosis could affect their ability to express their feelings and mood. Reported rates may therefore be an underestimation.^38^ We identified several studies, highlighting an important temporal relationship between brain tumor and suicidality within the first twelve months of diagnosis. For neuro-oncological providers, this window should be considered a higher risk, and we suggest the integration of basic mental health screening into early post-therapy follow-up visits. Novel interventions to improve the mental well-being of the patients might also prove beneficial.^46–52^ Studies that include the evaluation of psychiatric factors pre- and post-operation would also be recommended.^53^

While our knowledge of the etiology of SI and SA in brain tumor patients remains limited, a few important associations emerged from our literature synthesis: Tumor histopathology may influence SI and SA. For instance, a glioblastoma diagnosis was shown to be associated with an increased risk of suicide compared to other tumor types. The reasons for this are likely multifactorial, and we speculate they may reflect the poor prognosis, necessity for adjuvant therapy, often repeated dose regimens of steroids (known to confer mood side effects), and potential cerebral edema or predilection for supratentorial compartments.^35^ For low-grade glioma cases, treatment with surgery alone was associated with a higher risk of SI.^32,33^ Although the exact reason for this is unclear, the possibility of a delayed SI and SA, in the context of increased life expectancy, and the inevitable adjustments required in these patients, cannot be overlooked. Similarly, other studies have shown an increase in psychological comorbidities of glioma survivors who underwent surgery alone.^53,54^

There were mixed neuro-anatomical correlates to suicidality, with many studies lacking detailed descriptions of tumor locations. However, infratentorial procedures appeared to confer reduced risks of SI and SA based on our findings. Prior studies have suggested frontal lobe lesions can increase the risk of suicide,^55,56^ but this association was not found in the paper by Zhou and colleagues.^36^ Other reviewed studies also did not make a clear delineation of this relationship, which can be accounted for by the heterogeneity in population samples, assessment methods, and study designs. Future large-scale studies are required to address these questions; detailed neuroimaging and neuroanatomical region correlation to suicidality would be merited.

The relationship between anti-seizure medications and SI/ SA should be considered. Previous studies have shown that patients with risk factors, such as previous psychiatric disorders, traumatic brain injury history, substance misuse, and structural brain abnormality are at an elevated risk of SI/SA if prescribed levetiracetam.^57^ Future studies are required to investigate the relationship between these risk factors and increased SI/SA in brain tumor patients using anti-seizure medications. Furthermore, the side effects of steroids such as dexamethasone, which are commonly prescribed to brain tumor patients, have been noted prior,^58,59^ but their implications in psychiatric factors associated with SI and SA remain unexplored.

Suicidality was most often observed in persons with co-existing mental disorders, aligned with the manifestation of common biopsychosocial etiologies.^12,60,61^ Indeed, a history of depression is associated with SI in brain cancer survivors. These findings are consistent with general population studies, where it was shown depression was one of the primary predictors of suicidal behavior.^40,43^ From our identified studies, glioma patients with severe BDI scores had multiple domains of impairment, including physical, cognitive, and emotional health states.^38^ Furthermore, the male sex emerged as a risk factor, which is reflective of baseline population suicidality trends of completed suicides. Multiple other risk factors that were associated with risk for SI among childhood cancer survivors did not appear significant in this review, including lower education level, lower household income, younger age at diagnosis, recent unemployment, and unmarried status.^26^ Finally, refractory seizures were associated with SI.^33^ Such findings are consistent with other studies showing patients with epilepsy have an increased risk of suicide and reduced quality of life, and are relevant to the healthcare professionals managing these patients.^62–65^ Future studies are required to compare SI/SA in brain tumor patients to other brain pathologies, such as multiple sclerosis, traumatic brain injury, neurodegenerative diseases, and structural abnormalities, as well as the general cancer population to investigate the incidence and identify risk factors associated with SI/SA. Data beyond North America and Europe would also be merited to assess such trends in other continents.^66^ Last, concerning medical assistance in dying (MAiD) in brain tumor patients, there are differing opinions on its usage.^67^ It was shown that many patients opting for MAiD were at advanced and palliative-stage cancer^68^; however, brain tumor patients can be limited in their decision-making capacity.^69^ Future ethical and legal investigations are required to explore MAiD in patients’ refractory to psychological support.

## Limitations

While the total patient number reported in this review serves as the largest available pooled data examining suicidality in brain tumor patients, some limitations should be noted. First, there is heterogeneity in the articles reviewed with smaller studies identifying a more precise relationship between factors related to suicidal behavior and SI. Second, the two largest epidemiological studies from Saad et al.^35^ and Zhou et al.^36^ account for the vast majority of the subjects included with an overlapping population cohort from the SEER Program registries. Given that these researchers assessed completed suicides in brain tumor patients without reporting on unsuccessful attempts, the available data from the rates of SA derive from the remaining five studies. Furthermore, considering that five of the seven studies did not specify the rate of SAs resulting in mortality, it would be beneficial that future studies determine such a ratio, such that the experiences and psychological impacts on suicide survivors could be further studied and the risk factors leading to successful attempts be further identified. Third, the 203,832 patients (99.8%) from four studies were examined retrospectively, which could impact the risk of bias. Fourth, no study evaluated the risk of SI and SA before patients were diagnosed with brain tumors. Fifth, other factors such as personality traits, psychiatric support strategies provided, and cognitive functions can also influence the level of SI and SA, making such comparisons more challenging. Sixth, while studies recorded the presence of SI, such documentation did not provide standardized information on the severity and duration of such thoughts.

Psychological assessment and support are not a routine part of care in all neurosurgical units, and psychological follow-up is usually provided to patients who have complex medical and/or psychological needs, which can add further selection bias. Some studies had a long and varied interval between the patient’s diagnosis and/or underwent treatment and survey, which could potentially introduce recall bias. Future studies are required to compare the overall long-term SI and SA among brain tumor patients in an ideally prospective fashion. Despite such limitations, the current review can be a useful contribution to help understand suicidality among brain tumor patients and guide the configuration of healthcare and resources required.

## Conclusion

SI and SA are serious psychiatric conditions that appear more prevalent in patients with brain tumors. The number of brain tumor patients and survivors ideating and attempting suicide is concerning and highlights a potential adverse psychological consequence of experiencing a brain tumor diagnosis and treatment that should be recognized by neuro-oncological healthcare providers. A multidisciplinary approach is required to provide holistic follow-up care, especially including psychiatric risk assessment and support, for brain tumor patients. Potential associations between SI and SA with different risk factors require future large-scale, multi-center studies with long-term follow-up. Integration of psychiatric outcomes, perhaps at the same time points and alongside the quality of life measures, into prospective oncological trials would provide important neuro-anatomical and treatment associations with mental health outcomes. We suggest, at the very least, those patients with malignant brain tumors in the older age group should be screened regularly in the first year of their diagnoses and treatments for signs of SI. Currently, no specific tool exists for assessing SI among brain tumor patients. The development and validation of such psychiatric tools can help identify patients at risk for psychiatric referral and support. Also, dramatic SI fluctuation has been reported in previous studies.^70^ Therefore, investigations of the optimum frequency of follow-up with this population of patients are required. Additional studies concerning SI and SA during different phases of brain cancer diagnosis and treatment are needed to validate the risk factors described in this review and better understand the psychological burden of cancer diagnosis and treatment on patients.

## Supplementary Material

vdad058_suppl_Supplementary_Table_S1

## References

[1] Tran KB , Lang, JJ, Compton K, et al. The global burden of cancer attributable to risk factors, 2010-19: a systematic analysis for the Global Burden of Disease Study 2019. Lancet. 2022;400(10352):563–591. 35988567 10.1016/S0140-6736(22)01438-6 PMC9395583

[2] Kasper G , Hart S, Samuel N, Fox C, Das S. Anxiety and depression in patients with intracranial meningioma: a mixed methods analysis. BMC Psychol. 2022;10(1):93. 35395829 10.1186/s40359-022-00797-6 PMC8994241

[3] Poggi G , Liscio M, Galbiati S, et al. Brain tumors in children and adolescents: cognitive and psychological disorders at different ages. Psychooncology. 2005;14(5):386–395. 15386759 10.1002/pon.855

[4] Fehrenbach MK , Brock H, Mehnert-Theuerkauf A, Meixensberger J. Psychological distress in intracranial neoplasia: a comparison of patients with benign and malignant brain tumours. Front Psychol. 2021;12:1–9. 10.3389/fpsyg.2021.664235 PMC8418139 34489787

[5] Vyssoki B , Gleiss A, Rockett IR, et al. Suicide among 915,303 Austrian cancer patients: who is at risk? J Affect Disord. 2015;175:287–291. 25661393 10.1016/j.jad.2015.01.028

[6] Heinrich M , Hofmann L, Baurecht H, et al. Suicide risk and mortality among patients with cancer. Nat Med. 2022;28(4):852–859. 35347279 10.1038/s41591-022-01745-y

[7] Akechi T , Nakano T, Akizuki N, et al. Clinical factors associated with suicidality in cancer patients. Jpn J Clin Oncol. 2002;32(12):506–511. 12578898 10.1093/jjco/hyf106

[8] Ahn MH , Park S, Lee HB, et al. Suicide in cancer patients within the first year of diagnosis. Psychooncology. 2015;24(5):601–607. 25336020 10.1002/pon.3705

[9] Krysinska K , Lester D. Post-traumatic stress disorder and suicide risk: a systematic review. Arch Suicide Res. 2010;14(1):1–23. 20112140 10.1080/13811110903478997

[10] Turecki G , Brent DA. Suicide and suicidal behaviour. Lancet. 2016;387(10024):1227–1239. 26385066 10.1016/S0140-6736(15)00234-2 PMC5319859

[11] Ivey-Stephenson AZ , Crosby AE, Hoenig JM, Gyawali S, Park-Lee E, Hedden SL. Suicidal thoughts and behaviors among adults aged ≥18 years - United States, 2015-2019. MMWR Surveill Summ. 2022;71(1):1–19. 10.15585/mmwr.ss7101a1 PMC8736267 34990443

[12] Joshi K , Billick SB. Biopsychosocial causes of suicide and suicide prevention outcome studies in juvenile detention facilities: a review. Psychiatr Q. 2017;88(1):141–153. 27169893 10.1007/s11126-016-9434-2

[13] Turecki G. The molecular bases of the suicidal brain. Nat Rev Neurosci. 2014;15(12):802–816. 25354482 10.1038/nrn3839 PMC5293539

[14] Spoletini I , Gianni W, Caltagirone C, Madaio R, Repetto L, Spalletta G. Suicide and cancer: where do we go from here? Crit Rev Oncol/Hematol. 2011;78(3):206–219. 20605728 10.1016/j.critrevonc.2010.05.005

[15] Pranckeviciene A , Bunevicius A. Depression screening in patients with brain tumors: a review. CNS Oncol. 2015;4(2):71–78. 25768331 10.2217/cns.14.60 PMC6093018

[16] Shah SS , Dellarole A, Peterson EC, et al. Long-term psychiatric outcomes in pediatric brain tumor survivors. Childs Nerv Syst. 2015;31(5):653–663. 25726165 10.1007/s00381-015-2669-7

[17] Pelletier G , Verhoef MJ, Khatri N, Hagen N. Quality of life in brain tumor patients: the relative contributions of depression, fatigue, emotional distress, and existential issues. J Neurooncol. 2002;57(1):41–49. 12125966 10.1023/a:1015728825642

[18] Lupton A , Abu-Suwa H, Bolton GC, Golden C. The implications of brain tumors on aggressive behavior and suicidality: a review. Aggress Violent Behav. 2020;54:101416.

[19] Mansouri A , Boutet A, Elias G, et al. Lesion network mapping analysis identifies potential cause of postoperative depression in a case of cingulate low-grade glioma. World Neurosurg. 2020;133:278–282. 31606510 10.1016/j.wneu.2019.10.020

[20] Germann J , Zadeh G, Mansouri A, Kucharczyk W, Lozano AM, Boutet A. Untapped neuroimaging tools for neuro-oncology: connectomics and spatial transcriptomics. Cancers. 2022;14(3):464. 35158732 10.3390/cancers14030464 PMC8833690

[21] Bhanja D , Ba D, Tuohy K, et al. Association of Low-grade glioma diagnosis and management approach with mental health disorders: a MarketScan analysis 2005-2014. Cancers. 2022;14(6):1376. 35326529 10.3390/cancers14061376 PMC8946211

[22] Wyche M , Stokes BA, Shepherd JM, Kakulas BA, Ojeda VJ. High-grade cerebral neuroblastoma: a case study. Med J Aust. 1989;150(9):505–507. 2542739 10.5694/j.1326-5377.1989.tb136597.x

[23] Costanza A , Zenga F, Rudà R, et al. Suicidality in patients with brain tumors: a brief literature review with clinical exemplar. Medicina. 2020;56(12):725. 33371470 10.3390/medicina56120725 PMC7767493

[24] Moher D , Liberati A, Tetzlaff J, Altman DG. Preferred reporting items for systematic reviews and meta-analyses: the PRISMA statement. PLoS Med. 2009;6(7):e1000097. 19621072 10.1371/journal.pmed.1000097 PMC2707599

[25] World Health O. The ICD-10 Classification of Mental and Behavioural Disorders: Clinical Descriptions and Diagnostic Guidelines. Geneva: World Health Organization; 1992.

[26] Recklitis CJ , Diller LR, Li X, Najita J, Robison LL, Zeltzer L. Suicide ideation in adult survivors of childhood cancer: a report from the Childhood Cancer Survivor Study. J Clin Oncol. 2010;28(4):655–661. 19841325 10.1200/JCO.2009.22.8635 PMC2816000

[27] Lu D , Fall K, Sparén P, et al. Suicide and suicide attempt after a cancer diagnosis among young individuals. Ann Oncol. 2013;24(12):3112–3117. 24169626 10.1093/annonc/mdt415

[28] Gunnes MW , Lie RT, Bjørge T, et al. Suicide and violent deaths in survivors of cancer in childhood, adolescence and young adulthood-A national cohort study. Int J Cancer. 2017;140(3):575–580. 27750385 10.1002/ijc.30474

[29] Osazuwa-Peters N , Simpson MC, Zhao L, et al. Suicide risk among cancer survivors: head and neck versus other cancers. Cancer. 2018;124(20):4072–4079. 30335190 10.1002/cncr.31675

[30] Sharkey CM , Hardy KK, Gioia A, Weisman H, Walsh K. Suicidal ideation and executive functioning in pediatric cancer. Psychooncology. 2022;31(5):745–752. 34797956 10.1002/pon.5858

[31] Storm HH , Christensen N, Jensen OM. Suicides among Danish patients with cancer: 1971 to 1986. Cancer. 1992;69(6):1509–1512. 10.1002/1097-0142(19920315)69:6<1509::aid-cncr2820690632>3.0.co;2-j 1540887

[32] Turner CD , Chordas CA, Liptak CC, et al. Medical, psychological, cognitive and educational late-effects in pediatric low-grade glioma survivors treated with surgery only. Pediatr Blood Cancer. 2009;53(3):417–423. 19479971 10.1002/pbc.22081

[33] Brinkman TM , Liptak CC, Delaney BL, Chordas CA, Muriel AC, Manley PE. Suicide ideation in pediatric and adult survivors of childhood brain tumors. J Neurooncol. 2013;113(3):425–432. 23624716 10.1007/s11060-013-1130-6

[34] Lucas MS , Brawner BM, Hardie TL, et al. Assessing suicidal ideation and behaviors among survivors of childhood brain tumors and their mothers during sociobehavioral research. Oncol Nurs Forum. 2015;42(5):E319–329. 26302289 10.1188/15.ONF.42-05AP PMC4548293

[35] Saad AM , Elmatboly AM, Gad MM, et al. Association of brain cancer with risk of suicide. JAMA Netw Open. 2020;3(5):e203862. 32356882 10.1001/jamanetworkopen.2020.3862 PMC7195621

[36] Zhou Z , Jiang P, Zhang P, et al. Incidence, trend and risk factors associated with suicide among patients with malignant intracranial tumors: a surveillance, epidemiology, and end results analysis. Int J Clin Oncol. 2022;27(9):1386–1393. 35781641 10.1007/s10147-022-02206-9

[37] Pranckeviciene A , Tamasauskas S, Deltuva VP, Bunevicius R, Tamasauskas A, Bunevicius A. Suicidal ideation in patients undergoing brain tumor surgery: prevalence and risk factors. Support Care Cancer. 2016;24(7):2963–2970. 26868951 10.1007/s00520-016-3117-2

[38] Hickmann AK , Nadji-Ohl M, Haug M, et al. Suicidal ideation, depression, and health-related quality of life in patients with benign and malignant brain tumors: a prospective observational study in 83 patients. Acta Neurochir. 2016;158(9):1669–1682. 27318813 10.1007/s00701-016-2844-y

[39] Näher AF , Rummel-Kluge C, Hegerl U. Associations of suicide rates with socioeconomic status and social isolation: findings from longitudinal register and census data. Front Psychiatry. 2019;10:898. 31992995 10.3389/fpsyt.2019.00898 PMC6971176

[40] Crosby AE , Han B, Ortega LA, Parks SE, Gfroerer J. Suicidal thoughts and behaviors among adults aged ≥18 years--United States, 2008-2009. MMWR Surveill Summ. 2011;60(13):1–22. 22012169

[41] Naghavi M. Global, regional, and national burden of suicide mortality 1990 to 2016: systematic analysis for the Global Burden of Disease Study 2016. BMJ. 2019;364(8186):l94. 31339847 10.1136/bmj.l94 PMC6598639

[42] Nock MK , Hwang I, Sampson N, et al. Cross-national analysis of the associations among mental disorders and suicidal behavior: findings from the WHO World Mental Health Surveys. PLoS Med. 2009;6(8):e1000123. 19668361 10.1371/journal.pmed.1000123 PMC2717212

[43] Nock MK , Green JG, Hwang I, et al. Prevalence, correlates, and treatment of lifetime suicidal behavior among adolescents: results from the national comorbidity survey replication adolescent supplement. JAMA Psychiatry. 2013;70(3):300–310. 23303463 10.1001/2013.jamapsychiatry.55 PMC3886236

[44] Recklitis CJ , Lockwood RA, Rothwell MA, Diller LR. Suicidal ideation and attempts in adult survivors of childhood cancer. J Clin Oncol. 2006;24(24):3852–3857. 16921037 10.1200/JCO.2006.06.5409

[45] Lehuluante A , Fransson P. Are there specific health-related factors that can accentuate the risk of suicide among men with prostate cancer? Support Care Cancer. 2014;22(6):1673–1678. 24515278 10.1007/s00520-014-2150-2 PMC4008778

[46] Mofatteh M , Mashayekhi MS, Arfaie S, et al. Stress, anxiety, and depression associated with awake craniotomy: a systematic review. Neurosurgery. 2023;92(2):225–240. 36580643 10.1227/neu.0000000000002224 PMC9815094

[47] Price SJ , Whittle IR, Ashkan K, Grundy P, Cruickshank G. NICE guidance on the use of carmustine wafers in high-grade gliomas: a national study on variation in practice. Br J Neurosurg. 2012;26(3):331–335. 22482926 10.3109/02688697.2012.673651 PMC3432583

[48] Hurwitz M , Logan J, Bhangoo R, et al. Dramatherapy - A Unique Survivorship Intervention. 2012:3–3.

[49] Birks S , Altinkaya M , Altinkaya A, et al. Abstracts from the 2012 BNOS Conference. Neuro-Oncology. 2012;14(suppl_2):ii1–ii12.

[50] Mofatteh M , Mashayekhi MS, Arfaie S, et al. Augmented and virtual reality usage in awake craniotomy: a systematic review. Neurosurg Rev. 2022;46(1):19. 36529827 10.1007/s10143-022-01929-7 PMC9760592

[51] Mofatteh M. Neurosurgery and artificial intelligence. AIMS Neurosci. 2021;8(4):477–495. 34877400 10.3934/Neuroscience.2021025 PMC8611194

[52] Taeb S , Rostamzadeh D, Mafi S, et al. Update on mesenchymal stem cells: a crucial player in cancer immunotherapy. Curr Mol Med. 2023;23. 10.2174/1566524023666221226143814 36573062

[53] Meyer EA , Kieran MW. Psychological adjustment of “surgery-only” pediatric neuro-oncology patients: a retrospective analysis. Psychooncology. 2002;11(1):74–79. 11835594 10.1002/pon.553

[54] Armstrong GT , Conklin HM, Huang S, et al. Survival and long-term health and cognitive outcomes after low-grade glioma. Neuro-Oncology. 2011;13(2):223–234. 21177781 10.1093/neuonc/noq178 PMC3064628

[55] Turecki G , Ernst C, Jollant F, Labonté B, Mechawar N. The neurodevelopmental origins of suicidal behavior. Trends Neurosci. 2012;35(1):14–23. 22177979 10.1016/j.tins.2011.11.008

[56] Costanza A , D’Orta I, Perroud N, et al. Neurobiology of suicide: do biomarkers exist? Int J Legal Med. 2014;128(1):73–82. 23430141 10.1007/s00414-013-0835-6

[57] Esang M , Santos MG, Ahmed S. Levetiracetam and suicidality: a case report and literature review. Prim Care Companion CNS Disord. 2020;22(4):19nr02502. 10.4088/PCC.19nr02502 32731314

[58] Dietrich J , Rao K, Pastorino S, Kesari S. Corticosteroids in brain cancer patients: benefits and pitfalls. Expert Rev Clin Pharmacol. 2011;4(2):233–242. 21666852 10.1586/ecp.11.1 PMC3109638

[59] Hempen C , Weiss E, Hess CF. Dexamethasone treatment in patients with brain metastases and primary brain tumors: do the benefits outweigh the side-effects? Support Care Cancer. 2002;10(4):322–328. 12029432 10.1007/s00520-001-0333-0

[60] Mofatteh M. Risk factors associated with stress, anxiety, and depression among university undergraduate students. AIMS Public Health. 2021;8(1):36–65. 33575406 10.3934/publichealth.2021004 PMC7870388

[61] Rodríguez-Otero JE , Campos-Mouriño X, Meilán-Fernández D, Pintos-Bailón S, Cabo-Escribano G. Where is the social in the biopsychosocial model of suicide prevention? Int J Soc Psychiatry. 2022;68(7):1403–1410. 34533396 10.1177/00207640211027210

[62] Pompili M , Girardi P, Ruberto A, Tatarelli R. Suicide in the epilepsies: a meta-analytic investigation of 29 cohorts. Epilepsy Behav. 2005;7(2):305–310. 15996526 10.1016/j.yebeh.2005.05.010

[63] Bell GS , Gaitatzis A, Bell CL, Johnson AL, Sander JW. Suicide in people with epilepsy: how great is the risk? Epilepsia. 2009;50(8):1933–1942. 19453718 10.1111/j.1528-1167.2009.02106.x

[64] Tian N , Cui W, Zack M, Kobau R, Fowler KA, Hesdorffer DC. Suicide among people with epilepsy: a population-based analysis of data from the U.S. National Violent Death Reporting System, 17 states, 2003-2011. Epilepsy Behav. 2016;61:210–217. 27372961 10.1016/j.yebeh.2016.05.028 PMC6084424

[65] Tanti MJ , Marson AG, Chavredakis E, Jenkinson MD. The impact of epilepsy on the quality of life of patients with meningioma: a systematic review. Br J Neurosurg. 2016;30(1):23–28. 26982950 10.3109/02688697.2015.1080215

[66] Mofatteh M , Mashayekhi MS, Arfaie S, et al. Awake craniotomy in africa: a scoping review of literature and proposed solutions to tackle challenges. Neurosurgery. 2023; doi:10.1227/neu.0000000000002453 PMC10319364 36961213

[67] Climans SA , Mason WP, Variath C, Edelstein K, Bell JAH. Neuro-oncology clinicians’ attitudes and perspectives on medical assistance in dying. Can J Neurol Sci. 2021;48(6):772–778. 34321130 10.1017/cjn.2021.186

[68] Steck N , Egger M, Maessen M, Reisch T, Zwahlen M. Euthanasia and assisted suicide in selected European countries and US states: systematic literature review. Med Care. 2013;51(10):938–944. 23929402 10.1097/MLR.0b013e3182a0f427

[69] Chamberlain M. PALL-01. Physician assisted suicide in high grade gliomas: a university-based practice perspective. Neuro-Oncology. 2016;18(suppl_6):vi144–vi144.

[70] Harmer B , Lee S, Duong TVH, Saadabadi A. Suicidal ideation. StatPearls. StatPearls Publishing Copyright © 2023, StatPearls Publishing LLC; 2023.

