# Supplementary material for: Suicidal ideation and attempts in brain tumor patients and survivors: A systematic review
Source: Neurooncol Adv. 2023 May 12;5(1):vdad058. doi: 10.1093/noajnl/vdad058 (PMC10259251; doi:10.1093/noajnl/vdad058)
Supplement: vdad058_suppl_Supplementary_Table_S1 [file vdad058_suppl_supplementary_table_s1.docx]

**Supplementary Table 1.** Search Terms Used for Each Database

| **Database (search results number)** | **Search terms** |
| --- | --- |
| **PubMed** (880) | ("brain tumor"[All Fields] OR "brain neoplasms"[MeSH Terms] OR ("brain"[All Fields] AND "neoplasms"[All Fields]) OR "brain neoplasms"[All Fields] OR ("brain"[All Fields] AND "tumor"[All Fields]) OR "brain tumor"[All Fields] OR ("brain neoplasms"[MeSH Terms] OR ("brain"[All Fields] AND "neoplasms"[All Fields]) OR "brain neoplasms"[All Fields] OR ("brain"[All Fields] AND "cancer"[All Fields]) OR "brain cancer"[All Fields]) OR (("cranially"[All Fields] OR "skull"[MeSH Terms] OR "skull"[All Fields] OR "cranial"[All Fields]) AND ("cysts"[MeSH Terms] OR "cysts"[All Fields] OR "cyst"[All Fields] OR "neurofibroma"[MeSH Terms] OR "neurofibroma"[All Fields] OR "neurofibromas"[All Fields] OR "tumor s"[All Fields] OR "tumoral"[All Fields] OR "tumorous"[All Fields] OR "tumor"[All Fields] OR "neoplasms"[MeSH Terms] OR "neoplasms"[All Fields] OR "tumor"[All Fields] OR "tumor s"[All Fields] OR "tumoral"[All Fields] OR "tumorous"[All Fields] OR "tumors"[All Fields] OR "tumors"[All Fields])) OR (("cranially"[All Fields] OR "skull"[MeSH Terms] OR "skull"[All Fields] OR "cranial"[All Fields]) AND ("cancer s"[All Fields] OR "cancerated"[All Fields] OR "canceration"[All Fields] OR "cancerization"[All Fields] OR "cancerized"[All Fields] OR "cancerous"[All Fields] OR "neoplasms"[MeSH Terms] OR "neoplasms"[All Fields] OR "cancer"[All Fields] OR "cancers"[All Fields])) OR ("intra-cranial"[All Fields] AND ("cysts"[MeSH Terms] OR "cysts"[All Fields] OR "cyst"[All Fields] OR "neurofibroma"[MeSH Terms] OR "neurofibroma"[All Fields] OR "neurofibromas"[All Fields] OR "tumor s"[All Fields] OR "tumoral"[All Fields] OR "tumorous"[All Fields] OR "tumor"[All Fields] OR "neoplasms"[MeSH Terms] OR "neoplasms"[All Fields] OR "tumor"[All Fields] OR "tumor s"[All Fields] OR "tumoral"[All Fields] OR "tumorous"[All Fields] OR "tumors"[All Fields] OR "tumors"[All Fields])) OR ("intra-cranial"[All Fields] AND ("cancer s"[All Fields] OR "cancerated"[All Fields] OR "canceration"[All Fields] OR "cancerization"[All Fields] OR "cancerized"[All Fields] OR "cancerous"[All Fields] OR "neoplasms"[MeSH Terms] OR "neoplasms"[All Fields] OR "cancer"[All Fields] OR "cancers"[All Fields])) OR ("brain neoplasms"[MeSH Terms] OR ("brain"[All Fields] AND "neoplasms"[All Fields]) OR "brain neoplasms"[All Fields] OR ("brain"[All Fields] AND "metastasis"[All Fields]) OR "brain metastasis"[All Fields]) OR ("glioma"[MeSH Terms] OR "glioma"[All Fields] OR "gliomas"[All Fields] OR "glioma s"[All Fields]) OR ("glioblastoma"[MeSH Terms] OR "glioblastoma"[All Fields] OR "glioblastomas"[All Fields]) OR ("meningioma"[MeSH Terms] OR "meningioma"[All Fields] OR "meningiomas"[All Fields]) OR ("neuroma, acoustic"[MeSH Terms] OR ("neuroma"[All Fields] AND "acoustic"[All Fields]) OR "acoustic neuroma"[All Fields] OR ("acoustic"[All Fields] AND "neuroma"[All Fields])) OR ("astrocytoma"[MeSH Terms] OR "astrocytoma"[All Fields] OR "astrocytomas"[All Fields]) OR ("craniopharyngioma"[MeSH Terms] OR "craniopharyngioma"[All Fields] OR "craniopharyngiomas"[All Fields]) OR ("ependymoma"[MeSH Terms] OR "ependymoma"[All Fields] OR "ependymomas"[All Fields]) OR ("haemangioblastoma"[All Fields] OR "hemangioblastoma"[MeSH Terms] OR "hemangioblastoma"[All Fields] OR "haemangioblastomas"[All Fields] OR "hemangioblastomas"[All Fields]) OR (("lymphoma"[MeSH Terms] OR "lymphoma"[All Fields] OR "lymphomas"[All Fields] OR "lymphoma s"[All Fields]) AND ("brain"[MeSH Terms] OR "brain"[All Fields] OR "brains"[All Fields] OR "brain s"[All Fields])) OR ("medulloblastoma"[MeSH Terms] OR "medulloblastoma"[All Fields] OR "medulloblastomas"[All Fields]) OR ("meningioma"[MeSH Terms] OR "meningioma"[All Fields] OR "meningiomas"[All Fields]) OR ("oligodendroglioma"[MeSH Terms] OR "oligodendroglioma"[All Fields] OR "oligodendrogliomas"[All Fields]) OR ("pinealoma"[MeSH Terms] OR "pinealoma"[All Fields] OR ("pineal"[All Fields] AND "region"[All Fields] AND "tumors"[All Fields]) OR "pineal region tumors"[All Fields]) OR ("neuroma, acoustic"[MeSH Terms] OR ("neuroma"[All Fields] AND "acoustic"[All Fields]) OR "acoustic neuroma"[All Fields] OR ("vestibular"[All Fields] AND "schwannoma"[All Fields]) OR "vestibular schwannoma"[All Fields])) AND ("suicid"[All Fields] OR "suicidal ideation"[MeSH Terms] OR ("suicidal"[All Fields] AND "ideation"[All Fields]) OR "suicidal ideation"[All Fields] OR "suicidality"[All Fields] OR "suicidal"[All Fields] OR "suicidally"[All Fields] OR "suicidals"[All Fields] OR "suicide"[MeSH Terms] OR "suicide"[All Fields] OR "suicides"[All Fields] OR "suicide s"[All Fields] OR "suicided"[All Fields] OR "suiciders"[All Fields] OR ("parasuicidal"[All Fields] OR "parasuicidality"[All Fields] OR "suicide, attempted"[MeSH Terms] OR ("suicide"[All Fields] AND "attempted"[All Fields]) OR "attempted suicide"[All Fields] OR "parasuicide"[All Fields] OR "parasuicides"[All Fields]) OR ("self injurious behavior"[MeSH Terms] OR ("self injurious"[All Fields] AND "behavior"[All Fields]) OR "self injurious behavior"[All Fields] OR ("self"[All Fields] AND "harm"[All Fields]) OR "self harm"[All Fields]) OR ("self injurious behavior"[MeSH Terms] OR ("self injurious"[All Fields] AND "behavior"[All Fields]) OR "self injurious behavior"[All Fields] OR ("self"[All Fields] AND "harm"[All Fields]) OR "self harm"[All Fields]) OR "self-inflict"[All Fields] OR (("ego"[MeSH Terms] OR "ego"[All Fields] OR "self"[All Fields]) AND ("inflict"[All Fields] OR "inflicted"[All Fields] OR "inflicting"[All Fields] OR "infliction"[All Fields] OR "inflicts"[All Fields])) OR "self-injur"[All Fields] OR (("ego"[MeSH Terms] OR "ego"[All Fields] OR "self"[All Fields]) AND "injur"[All Fields]) OR "self-cut"[All Fields] OR (("ego"[MeSH Terms] OR "ego"[All Fields] OR "self"[All Fields]) AND "cut"[All Fields]) OR (("ego"[MeSH Terms] OR "ego"[All Fields] OR "self"[All Fields]) AND "immolate"[All Fields]) OR "self-destruct"[All Fields] OR (("ego"[MeSH Terms] OR "ego"[All Fields] OR "self"[All Fields]) AND ("destruct"[All Fields] OR "destructed"[All Fields] OR "destructing"[All Fields] OR "destruction"[All Fields] OR "destructions"[All Fields] OR "destructive"[All Fields] OR "destructively"[All Fields] OR "destructs"[All Fields])) OR "self-poison"[All Fields] OR (("ego"[MeSH Terms] OR "ego"[All Fields] OR "self"[All Fields]) AND ("poisoned"[All Fields] OR "poisoning"[MeSH Terms] OR "poisoning"[All Fields] OR "poisonings"[All Fields] OR "poisoning"[MeSH Subheading] OR "poisonous"[All Fields] OR "poisons"[Pharmacological Action] OR "poisons"[MeSH Terms] OR "poisons"[All Fields] OR "poison"[All Fields])) OR "overdos"[All Fields]) |
| **Scopus** (879) | TITLE-ABS-KEY ( brain  AND tumor  OR  brain  AND cancer  OR  cranial  AND tumor  OR  cranial  AND cancer  OR  intra-cranial  AND tumor  OR  intra-cranial  AND cancer  OR  brain  AND metastasis  OR  glioma  OR  glioblastoma  OR  meningioma )  AND  ( suicide  OR  self-harm  OR  self-injury  OR  self-cut ) |
| **Web of Science** (239) | (TI=(( brain tumor OR brain cancer OR cranial tumor OR cranial cancer OR intra-cranial tumor OR intra-cranial cancer OR brain metastasis) AND (suicide OR self-harm OR self-injury))) OR AB=(( brain tumor OR brain cancer OR cranial tumor OR cranial cancer OR intra-cranial tumor OR intra-cranial cancer OR brain metastasis) AND (suicide OR self-harm OR self-injury)) |
